# Interpretable machine learning framework for peptide lead optimization in ultralarge design spaces

**DOI:** 10.1038/s44386-026-00069-y

**Published:** 2026-10-12

**Authors:** Yixiang Mao, Ruibo Zhang, Robert Sheridan, Dan Sindhikara, Dipannita Kalyani, Li Xiao, Amy B. McCracken, Bryant Kim, Shasha Feng, Tianxiong Mi, Christopher W. Plummer, Jennifer M. Johnston, Liying Zhang

**Affiliations:** 1 Department of Electrical and Computer Engineering, Texas Tech University, Lubbock, TX USA; 2 Discovery Chemistry, Merck & Co., Inc., Rahway, NJ USA

**Keywords:** Chemistry, Computational biology and bioinformatics, Drug discovery, Mathematics and computing

## Abstract

In modern peptide drug discovery, the incorporation of noncanonical amino acids (ncAAs) has become a powerful strategy to improve potency and key developability properties, including stability, solubility, and oral bioavailability. This shift has expanded peptide chemical diversity and design spaces well beyond natural residues, creating fundamental challenges for predictive methods (e.g., quantitative structure-activity relationship modeling) used in lead optimization: limited transferability from natural residues to novel ncAAs, limited interpretability, and poor scalability to ultralarge peptide spaces (>10^50^). We present PepOpt, an interpretable machine learning framework that unifies molecule-based and sequence-based peptide representations in linear models and exploits dynamic programming to efficiently explore ultralarge peptide spaces. PepOpt achieves high predictive accuracy and interpretability, supports training and inference across diverse ncAAs, including ncAAs absent from the training set whose constituent substructures are represented in the training data. Its linear structure decomposes predictions into residue-specific contributions, enabling systematic optimization without exhaustive sequence enumeration. Notably, PepOpt is inherently capable of multi-parameter optimization without specialized modifications. Together, these capabilities position PepOpt as a valuable platform for prediction, interpretation, ideation, and rapid exploration in ncAA-rich ultralarge peptide chemical spaces, accelerating the rational design of novel peptide therapeutics.

## Introduction

Peptides are an attractive therapeutic modality due to their unique advantages, including high binding affinity, specificity, and the ability to engage targets traditionally considered “undruggable” for small molecules (e.g., shallow binding pockets and protein-protein interfaces)^1,2^. However, peptides composed primarily of the 20 natural (canonical) amino acids (AAs) have poor pharmacokinetic properties, such as low gastrointestinal stability and membrane permeability, which limit their success as drug candidates^2–4^. Incorporating noncanonical amino acids (ncAAs) provides a means to fine-tune both potency and pharmacokinetic properties. Meanwhile, advances in technologies, such as mRNA display^5^ and late-stage functionalization (LSF)^3^, have further enabled ncAA integrations. Recent examples, such as Enlicitide by Merck & Co., Inc., Rahway, NJ, USA^6^ and Icotrokinra by Johnson & Johnson^7^, have demonstrated the therapeutic potential of ncAA-containing peptides.

The incorporation of ncAAs provides unprecedented flexibility in peptide sequence design and astronomically large design spaces. Navigating such spaces to optimize potency and properties remains a major challenge, highlighting the need for robust computational frameworks tailored to peptides containing ncAAs. Quantitative structure-activity relationship (QSAR) modeling is widely used in computer-aided drug design^8–10^. QSAR models require molecular descriptors and a machine learning (ML) method that relates the descriptors to the property of interest^11,12^. For peptide design, linear^13^, nonlinear^14^, and deep-learning methods^15–18^ have been explored. Broadly, peptide models fall into two families:

1. Molecule-based approaches that treat peptides as larger-than-usual whole molecules and use the descriptors and methods originally developed for small-molecule chemistry^17,19^. Peptides can be represented with global descriptors, molecular graphs^18^, or fragments as tokens^20,21^. These approaches do not naturally preserve explicit positional information, which limits residue-level interpretability and hinders their broader application in peptide design.
  Also note that fragment-based vocabularies are prone to out-of-vocabulary issues when extrapolating to novel or chemically modified residues from peptide projects. Moreover, unlike proteins—whose sequences reflect strong evolutionary constraints and shared grammar—peptide therapeutics originate from synthetically generated hits, which can limit the effectiveness of masked pretrained models.
2. Sequence-based approaches that encode residues at each position, such as one-hot encoding (OHE) used in the Free-Wilson (FW) modeling^22,23^, or assign physicochemical properties to individual residues (e.g., Zscale descriptors^24^). In these methods, positional information is explicitly preserved. Although widely used in peptide modeling, these methods lack explicit structural information and struggle to apply to novel ncAAs absent from the training data. A promising alternative, which forms a core element of our approach, is to assign substructure descriptors to individual residues. This strategy addresses the aforementioned limitations but remains relatively uncommon in the literature.

A further limitation of conventional QSAR models is that they typically require enumerating and scoring full sequences to identify the best-scored candidates, while the sizes of ncAA peptide design spaces make exhaustive search infeasible. For instance, with ~4000 monomers, the number of possible 14-mer macrocyclic peptides is on the order of 10^50^, which is well beyond typical small-molecule virtual libraries^25^. Heuristic strategies, such as Thompson sampling (TS) and genetic algorithms (GAs)^25–28^, could be used to effectively reduce the search burden. However, peptide-scale spaces remain orders of magnitude larger than where these methods are applied^26^, and in such complex spaces, stochastic searches inevitably risk converging to local minima^29^.

In this work, we introduce PepOpt, a machine-learning framework that jointly addresses key requirements for computer-aided peptide design: interpretability, predictivity, applicability to novel ncAAs, and scalability to ultralarge design spaces. PepOpt unifies molecule-based (structure-aware) and sequence-based (position-aware) representations through position-specific substructure descriptors, and pairs them with a linear regression backbone whose additive structure yields residue- and substructure-level attributions. Because monomer contributions are calculated from substructures, PepOpt transfers to ncAAs absent from training whose constituent substructures overlap with those observed during training, and, coupled with dynamic programming (DP), enables deterministic exploration of design spaces containing more than 10^50^ candidate sequences. This manuscript describes the framework, benchmarks it against linear and nonlinear baselines across multiple internal peptide series, and demonstrates its use for SAR elucidation, lead optimization, and multi-parameter optimization (MPO).

## Results

### Overview of the PepOpt framework

**Fig. 1:**
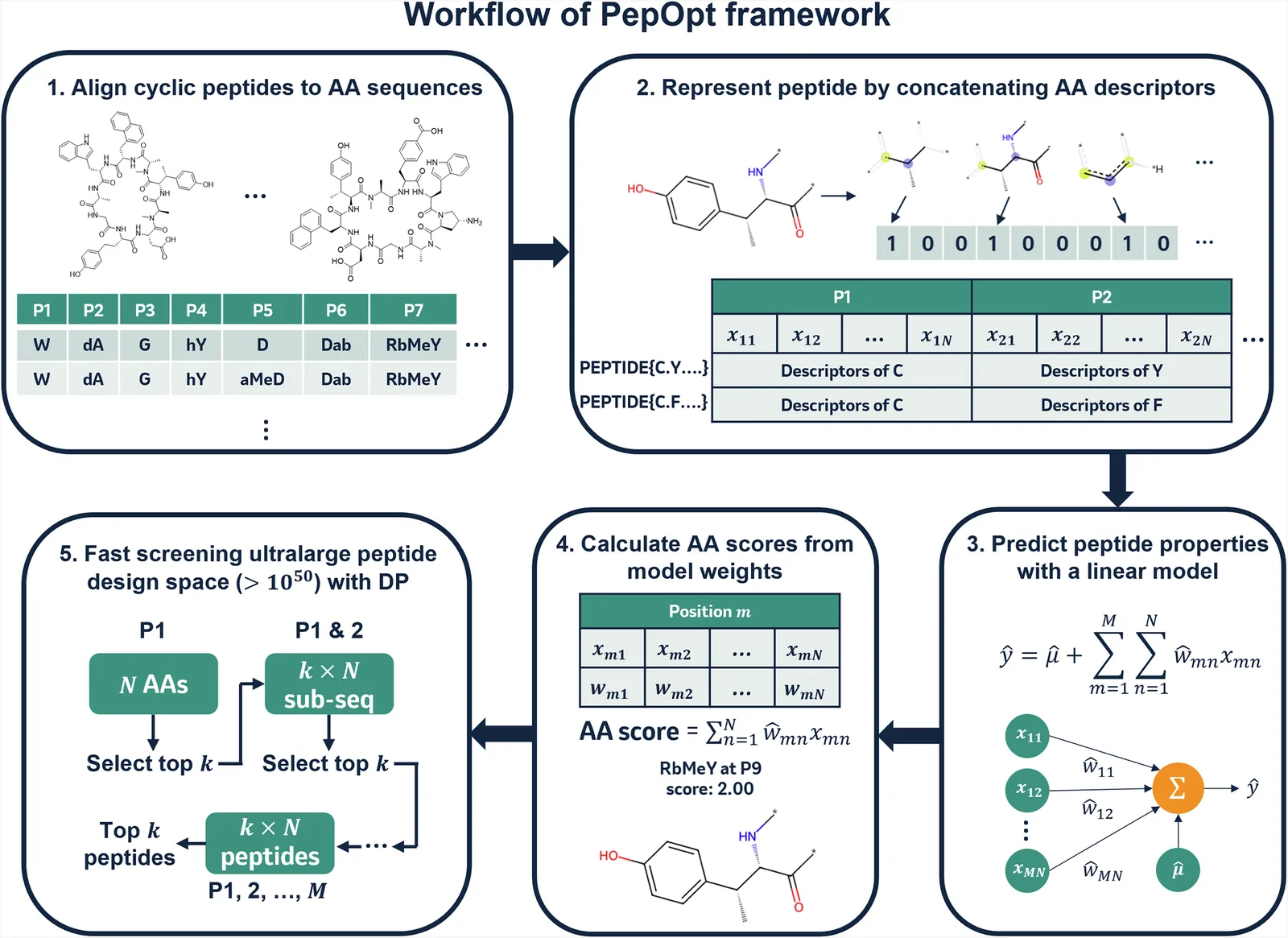
Overview of the PepOpt workflow. DP dynamic programming.

PepOpt comprises four key components: (1) peptide alignment to enable sequence-based peptide representations; (2) generation of position-specific fingerprints using computationally efficient and broadly accessible descriptor generators compatible with both canonical and noncanonical AAs; (3) a linear model to ensure model interpretability; and (4) DP to enable an efficient search for high-potential peptide candidates from ultralarge design spaces. Figure 1 illustrates the overall workflow of the PepOpt framework with detailed descriptions in the Methods section.

### Impacts on PepOpt model performance from various combinations of molecular descriptors and models

For components (1) and (2), we evaluated four open-source molecular representations and six linear models, resulting in 48 descriptor-linear model combinations. Full details of the molecular representations and model formulations are provided in the Methods section. All methods were evaluated on datasets of 11 distinct peptide series from 5 proprietary drug discovery projects. Series are labelled A-1, A-2, …, E-1, where the letter denotes the target and the number indexes the series within that target. The number of peptides in each series varies from 415 to 2013, and the length of peptides varies from 14 to 17. More characteristics of each series are summarized in Table S1. Peptide binding affinity, the primary property used in experiments, was measured and calculated as 9 − *log*_10_(*EC*50), where EC50 is expressed in nanomolar units. Mixing results from different assays can be risky^30^, and in our dataset, EC50 values in each series are collected from the same type of assay.

**Fig. 2:**
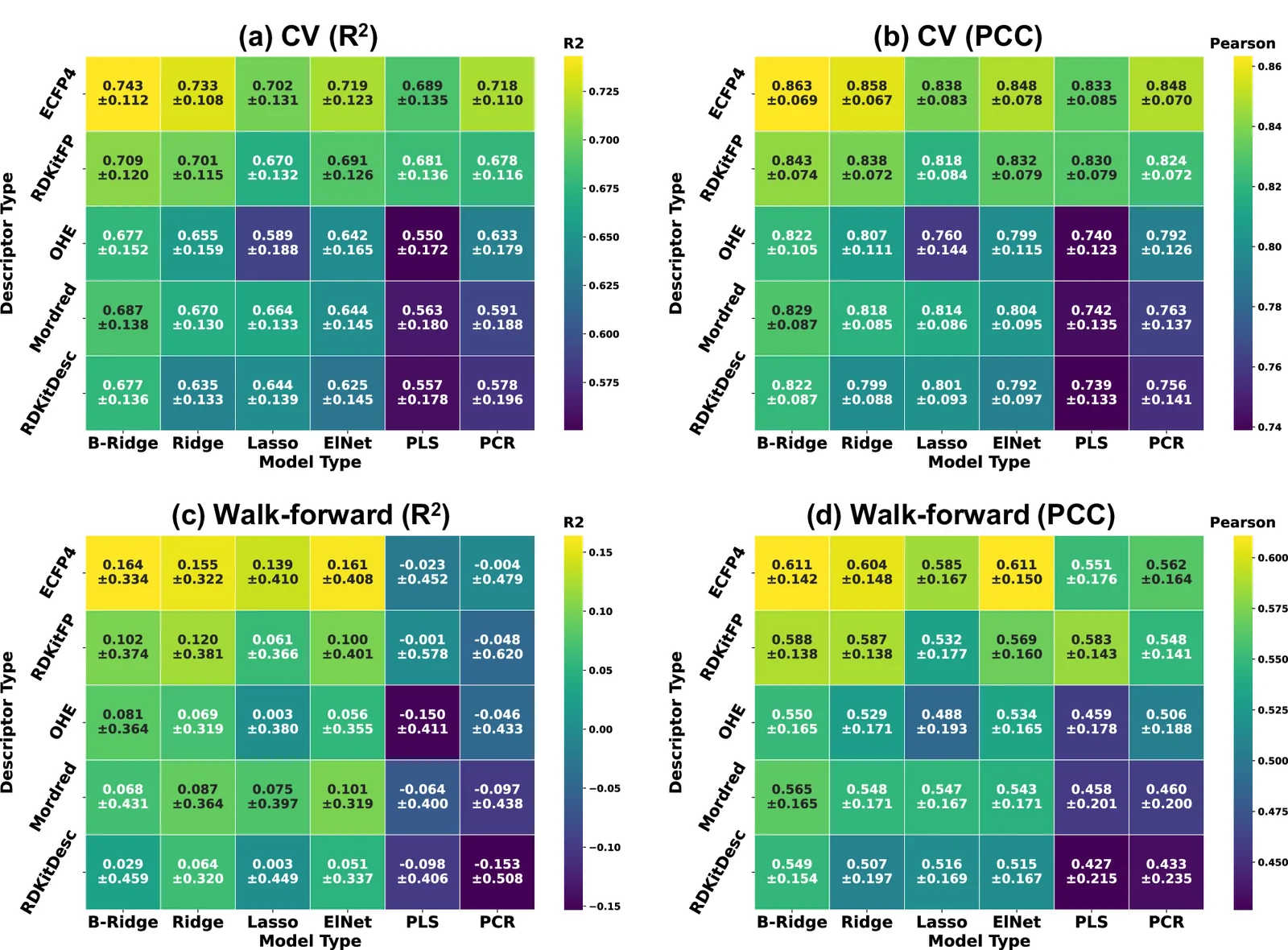
Benchmarking various descriptor-model combinations to optimize the PepOpt framework configurations. Peptide potency prediction with 5-fold random cross-validation (**a**, **b**) and walk-forward validation (**c**, **d**). The heatmaps visualize averaged performance over split folds and peptide series for *R*^2^ (**a**, **c**) and PCC (**b**, **d**). Cell annotations report the mean and standard deviation values. Additional results, including time-split validation and Spearman correlation, are available in Fig. S2. OHE one-hot encoding, RDKitDesc RDKit descriptors, B-Ridge Bayesian Ridge, ElNet Elastic Net, PLS partial least squares, PCR principal component regression.

Figure 2 comprehensively shows the performance of descriptor-linear model combinations for peptide potency prediction with 5-fold random cross-validation (top row) and walk-forward validation (bottom row). The heatmaps visualize model performance averaged over all split folds and peptide series for both *R*^2^ (left column) and Pearson correlation coefficient (PCC) (right column). Cell annotations report the mean and standard deviation values. Additional results, including time-split validation and Spearman correlation, are available in Fig. S2.

Although absolute performance varies across different splitting types and performance metrics, a consistent pattern emerges from the heatmaps. The best-performing combinations (bright yellow) are concentrated in the top-left, while the worst-performing combinations (dark blue) are in the bottom-right. The top-left corner corresponds to combinations of binary fingerprints (ECFP4 and RDKitFP) with regularized linear models (i.e., B-Ridge, Ridge, Lasso, and ElNet). In contrast, the bottom-right corner represents combinations of physical property descriptors (Mordred and RDKitDesc) and linear models with dimension reduction (i.e., PLS and PCR).

To identify the top-performing combinations, we ranked them for each splitting type and performance metric (detailed ranks are in Fig. S3). We then calculated an average rank for each combination across all conditions to determine its overall effectiveness. The five combinations with the best (lowest) average ranks are shown in Fig. S3. The combination of ECFP4 with B-Ridge demonstrated the best performance in eight out of the nine cases (three splitting types times three performance metrics), resulting in the lowest average rank of 1.1. The other four top-performing combinations are: ECFP4 + Ridge (avg. rank 2.4), ECFP4 + ElNet (avg. rank 2.7), RDKit + B-Ridge (avg. rank 5.4), and RDKitFP + Ridge (avg. rank 6.4).

Given its consistently superior performance across nearly all evaluation conditions, coupled with the inherent advantages of B-Ridge over standard Ridge, the combination of ECFP4 and B-Ridge was selected as the optimal configuration for the PepOpt framework.

### Comparing PepOpt to nonlinear QSAR models

**Fig. 3:**
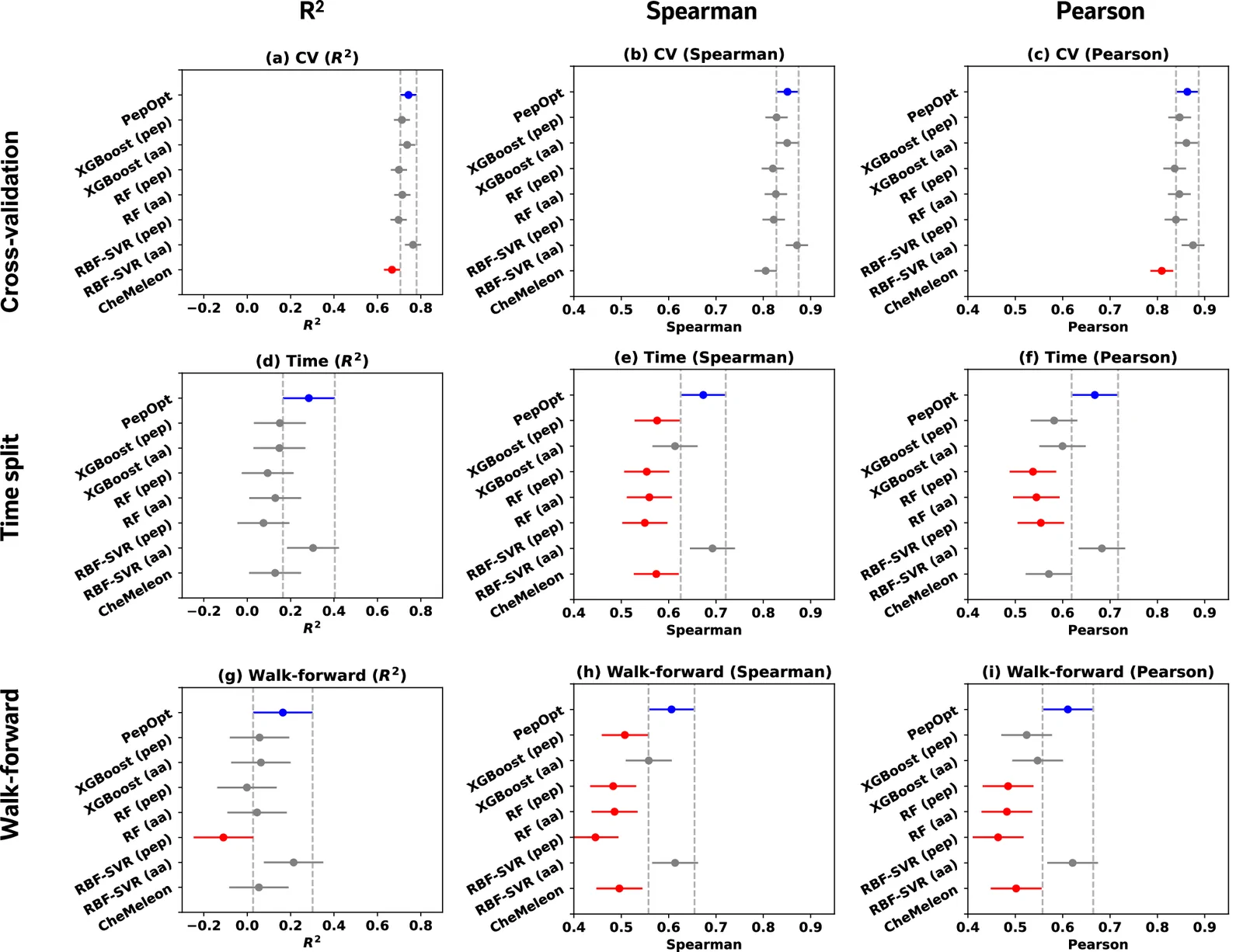
Comparison of the optimized PepOpt framework (PepOpt) against nonlinear models: RBF-SVR, RF, XGBoost, and CheMeleon across train-test split types and performance metrics. **a**–**c** random cross-validation; (**d**–**f**) time split; (**g**–**i**) walk-forward split. For SVR, RF, and XGBoost, two peptide representations were evaluated: (1) (aa), formed by concatenating ECFP4 fingerprints of AAs across the peptide sequence (as used in PepOpt), and (2) (pep), the count-based ECFP4 fingerprint computed for the whole peptide. Tukey’s Honestly Significant Difference (HSD) test was used to determine if the performance of PepOpt was significantly (*p* < 0.05) different from the nonlinear models. The dots represent the mean performance over split folds and peptide series, and the bars show the confidence intervals. Colors indicate statistical comparison to PepOpt: blue, performance of PepOpt; gray, statistically equivalent to PepOpt; red, significantly different from PepOpt.

To further assess its predictive power, we compared PepOpt against several nonlinear models, including random forest (RF), support vector regression with an RBF kernel (RBF-SVR), gradient-boosting machine (XGBoost), and CheMeleon (a Chemprop-based pretrained message passing neural network model). For peptide-specific pretrained models, we compared Pepland, a pretrained multi-view heterogeneous graph neural network connected with a two-layer multilayer perceptron (MLP) as a prediction head. For RF, SVR, and XGBoost, we provided both whole-molecule fingerprints (noted as pep) and concatenated monomer fingerprints (noted as aa). The predictive performance of these models is shown in Fig. 3. For each splitting type and performance metric, following the proposed comparison method in ref. ^31^, Tukey’s Honestly Significant Difference test^32^ was used to determine if the performance of PepOpt was significantly (*p* < 0.05) different from that of the nonlinear models. Detailed metrics are provided in Table S2. In this experiment, Pepland-MLP exhibits significantly lower scores, which are not reported in Fig. 3 to better illustrate the performances of other models.

As shown in Fig. 3, PepOpt consistently outperforms other QSAR models and shows comparable performance to the best-performing nonlinear model, RBF-SVR (aa), placing it among the best across splitting types and performance metrics. Furthermore, for the two temporal splitting types, PepOpt significantly outperformed most competitive models except for XGBoost (aa) and RBF-SVR (aa) in both Spearman and Pearson correlations. These results suggest that PepOpt achieves predictive performance that is comparable to or superior to the evaluated nonlinear models for ncAA-containing peptide potency prediction. Another pattern we observed is the overall superior performance of monomer fingerprints over whole-molecule fingerprints. This indicates the importance of positional information in peptide QSAR modeling.

We next investigated model performance under different evaluation schemes. For the 5-fold random CV, all models showed strong predictive performance, with *R*^2^ values from 0.67 to 0.76, Spearman correlations from 0.80 to 0.87, and PCC values from 0.81 to 0.88. In contrast, when evaluated under the two temporal splitting strategies, all models exhibited a substantial drop in *R*^2^, indicating greater difficulty in predicting exact potency values. This is not at all surprising; the literature often reports that random splits are more optimistic than time-based splits. However, PepOpt maintained decent performance in Spearman and Pearson correlations, ranging from 0.61 to 0.67, suggesting that it effectively captured the rank-ordering and linear trends of peptide potency. This capability is essential for prioritizing high-potential candidates in a discovery campaign.

Figure S4 displays the series-wise PCC for each model across different splitting types. PepOpt consistently demonstrated top-tier performance across diverse peptide series with varying lengths, targets, and sample sizes, indicating its robustness and generalizability.

Overall, PepOpt demonstrates highly competitive predictive performance, especially in temporal splitting types, reinforcing its utility for peptide SAR elucidation and lead optimization.

### Peptide SAR elucidation and lead optimization with the PepOpt framework

This section provides illustrative examples of using PepOpt to elucidate peptide SAR and guide optimization.

**Fig. 4:**
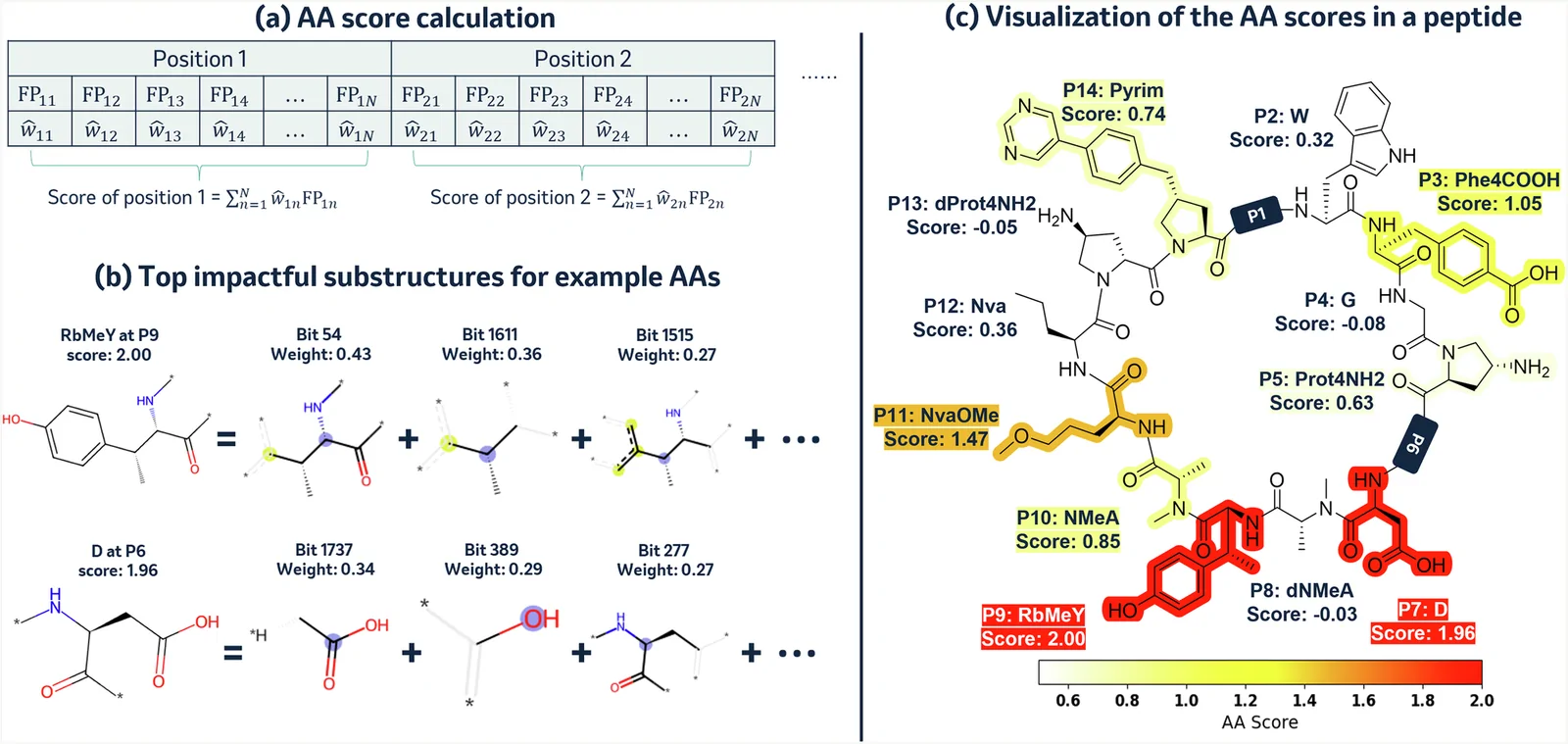
Illustration of peptide SAR deconvoluted by the model trained on series A-2. **a** At each position, the score of an AA is calculated as the sum over element-wise products of its FPs and the model coefficients associated with that position. **b** Top impactful FPs and their associated substructures for two example AAs: RbMeY at position 9 and D at position 6. **c** Visualization of the AA scores in an example peptide, showing how individual AAs directly contribute to the predicted potency. Higher scores indicate stronger positive contributions to potency. The color bar is cut off at 0.5 to highlight the most impactful AAs. Chemical structures are drawn with ChemDraw.

Since PepOpt employs a linear model, the effect of each FP bit (or equivalently, each molecular substructure) on potency can be directly assessed by its corresponding model coefficient. The sign of the model coefficient indicates the direction of the effect (positive or negative), while its magnitude reflects the strength of that effect. In addition to this substructure-level analysis, we can also evaluate the effect of each AA by calculating an AA score. As illustrated in Fig. 4a, the score for an AA at a specific position is calculated as the sum over the element-wise product of its FPs and the corresponding model coefficients for that position. These AA scores allow us to directly assess the position-specific effect of individual AAs on potency.

Figure 4b presents two examples of high-scoring AAs from an A-2 peptide: RbMeY at position 9 (P9) with a score of 2.00, and D at position 6 (P6) with a score of 1.96. For these highest-scoring AAs, the three highest-ranking substructures (FP bits) were highlighted as the features most impactful toward potency within the framework of the learned model. Interestingly, in the case of RbMeY, the top three substructures are all from its R-beta-methyl group, indicating that the presence of this group at P9 plays a critical role in enhancing potency. Furthermore, the aromatic attachment point to the phenol ring is highlighted, suggesting that the model assigns greater importance to this linkage relative to the amino-acid backbone. In contrast, for D, the selected substructures span both the backbone and the carboxylic acid moiety, highlighting a greater reliance on this functional group as a major contributor to its importance at P6. Encouragingly, the importance of the R-beta-methyl substructure at P9 was confirmed by experimental data, suggesting that PepOpt can successfully capture and reveal the important substructures for potency.

Figure 4c visualizes AA scores for an example peptide interpreted by a model trained from series A-2. It can be observed that AAs at different positions have varying contributions to the predicted potency. For instance, RbMeY at P9 and D at P7 have high scores, indicating their strong boosting effect on potency. In contrast, G at P4, dNMeA at P8, and dProt4NH2 at P13 show scores close to zero, suggesting that these three AAs may have minimal impact on potency.

A key feature of PepOpt relative to the FW model with the OHE representation is that AA scores can be calculated not only for AAs present in the training set but also for any AA for which valid FPs can be calculated. This capability allows for the virtual screening of a large AA library to identify novel and high-potential candidates. For any given position, we can calculate scores for all AAs in the library and rank them to select top candidates for the next round of experimental validation.

**Fig. 5:**
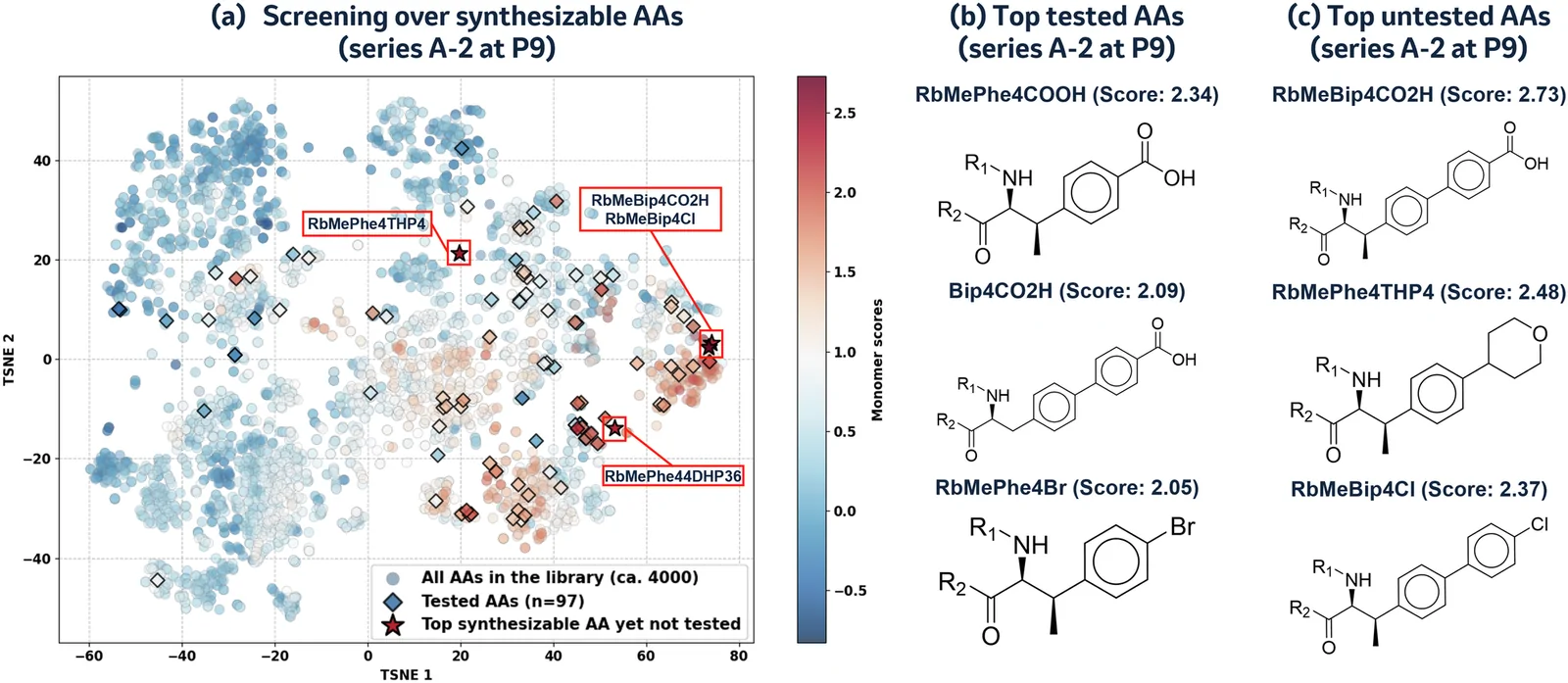
Identification of promising synthesizable AAs that have not been tested for series A-2 at position 9. **a** Visualization of the scores for ~4000 AAs in a proprietary virtual library. The AA scores were calculated with the model trained on series A-2 at position 9. Diamond symbols mark AAs previously tested in series A-2 at position 9. Star symbols highlight three untested AAs with scores exceeding that of the best tested AA (RbMePhe4COOH, score = 2.34). **b** Structures of the top-scoring tested AAs. **c** Structures of the top-scoring untested AAs. Chemical structures are drawn with ChemDraw.

To demonstrate this capability, we use the example of finding alternative amino acids for RbMeY at position P9 in series A-2, aiming for equal or improved potency based on model predictions. Figure 5a depicts the chemical space of ca. 4000 candidate monomers, among which 97 monomers have been selected for this position in synthesis and experimentally tested for potency. We calculated scores for all candidate monomers, whether used in synthesis or not, and highlighted the top-scoring ones in (b) and (c). This shows how the model makes predictions on unseen monomers leveraging learned substructural weights. As shown in (c), the model identifies monomers that never appeared in the training set with higher scores than synthesized ones: RbMeBip4CO2H (score = 2.73), RbMePhe4THP4 (score = 2.48), RbMeBip4Cl (score = 2.37). Notably, all suggestions feature the R-beta-methyl group that was identified as a key modification, plus the key motifs that are associated with higher potency in the training set.

## Efficient exploration of ultralarge peptide design spaces with PepOpt

Peptide optimization is commonly performed by mutating a lead peptide at selected positions, typically using a fixed set of available AA building blocks to ensure synthesizability. The introduction of ncAAs dramatically expands the accessible design space, which makes exhaustive enumeration infeasible. For example, mutating only four positions from a pool of ~4000 monomers already yields >10^14^ sequences. Special methods are required for such massive spaces.

In PepOpt, efficient traversal of ultralarge design spaces is enabled by scoring individual monomers and DP. Although monomers at different positions might interact with each other in reality, PepOpt assumes additivity, i.e., a peptide’s predicted activity equals the sum of scores of all AA components. Under this assumption, any subsequence of the best-predicted peptide must be locally optimal, satisfying the DP principle and allowing deterministic search. Consider the task of finding the top *k* sequences from a search space defined by mutating *M* positions, each with *N* possible AAs. For each mutation step *i*(*i* = 1, 2, …*M*), the resulting scores only depend on step *i* and that of *i* − 1. At each step *i*, the top *k* subsequences from step *i* − 1 are paired with all *N* candidate AAs from step *i*, resulting in *k* × *N* combinations, which are then sorted to find the next top *k* subsequences. This process of extension and selection is repeated for all *M* mutated positions. Therefore, the time complexity of DP is $O(MkN\cdot \log (kN))$. Upon reaching the final mutated position, the algorithm returns the top *k* complete peptide sequences with the highest predicted potency in the entire design space, given the mutated positions and the AA library. Since it is a deterministic approach, the choice of starting position does not affect the final results. Also, it can be applied to any subset of positions, up to and including the entire sequence.

To illustrate the contribution of DP, we compared its efficiency and solution quality with those of a GA search, and estimated the computational cost of exhaustive enumeration coupled with nonlinear predictive models. Using the A-2 series of 14-mer peptides in the training set, we trained a Bayesian Ridge regression model with concatenated monomer fingerprints and searched for optimal sequences across two design spaces: (1) the space spanned by monomers present in training (>10^20^ sequences), and (2) a broader space defined by a library of ~4,000 monomers (10^50^ possible sequences).

In GA searches, sequences were iteratively improved over successive generations. In space (1), GA converged after 29 generations with a maximal predicted score of 10.928 and no further improvement from the 29th to the 50th generation; in space (2), scores continued to increase through the 50th generation without clear convergence, reaching 10.517. DP identified higher-scoring sequences in both spaces (11.576 and 12.143, respectively) and deterministically found the globally optimal sequence regardless of *k*. To assess computational performance, we measured wall-clock time to enumerate the top 1000 sequences via DP; a detailed DP-GA comparison is provided in Table 1. We ran ten random GA search campaigns on the 10^20^ and 10^50^ spaces, respectively, and plotted their highest score per generation (Fig. S5), showing how the sequences evolve during GA. These GA campaigns show similar trends. Collectively, these results highlight the fundamental differences between the two approaches: GA relies on stochastic sampling and can converge to different local optima depending on initialization and search trajectory, while DP guarantees optimality within the model formulation with predictable computational cost.

**Table 1:** Time complexity for searching the highest-scoring sequences in design spaces

Search methods	Design space with used AAs (10^20^ sequences)	Ultralarge design space with novel AAs (10^50^ sequences)
DP^a^ $O(M\cdot k\cdot N\cdot \log (kN))$	2.187 s	174.4 s
GA	12.73 h	11.63 h
Exhaustive search with model:	Time consumption estimated from model prediction time. Unit: CPU years	
RF	1.8 × 10^8^	3.7 × 10^38^
XGBoost	1.3 × 10^9^	1.1 × 10^39^
RBF-SVR	1.3 × 10^11^	1.2 × 10^41^
FW	6.3 × 10^6^	N/A^b^

^a^Time complexity analysis: *M* modifiable positions, *N*-membered monomer library, and finding the top *k* peptides.

^b^Not applicable: the OHE FW model cannot extrapolate to AAs absent from training data.

As discussed earlier, exhaustive searching coupled with predictive models is computationally prohibitive. We estimated the computational time based on per-prediction timings (Fig. S6), and the estimated time to screen the 10^20^ space ranges from 6.3 × 10^6^ (FW) to 1.3 × 10^11^ (RBF-SVR) CPU years; for the ultralarge space, estimates rise to ~10^38^ (RF and XGBoost) and ~10^41^(RBF-SVR) CPU years. These estimations are based on running predictions on small, enumerated sets, without considering the additional costs for I/O, sorting, and storage.

## Using PepOpt for multi-parameter optimization

**Fig. 6:**
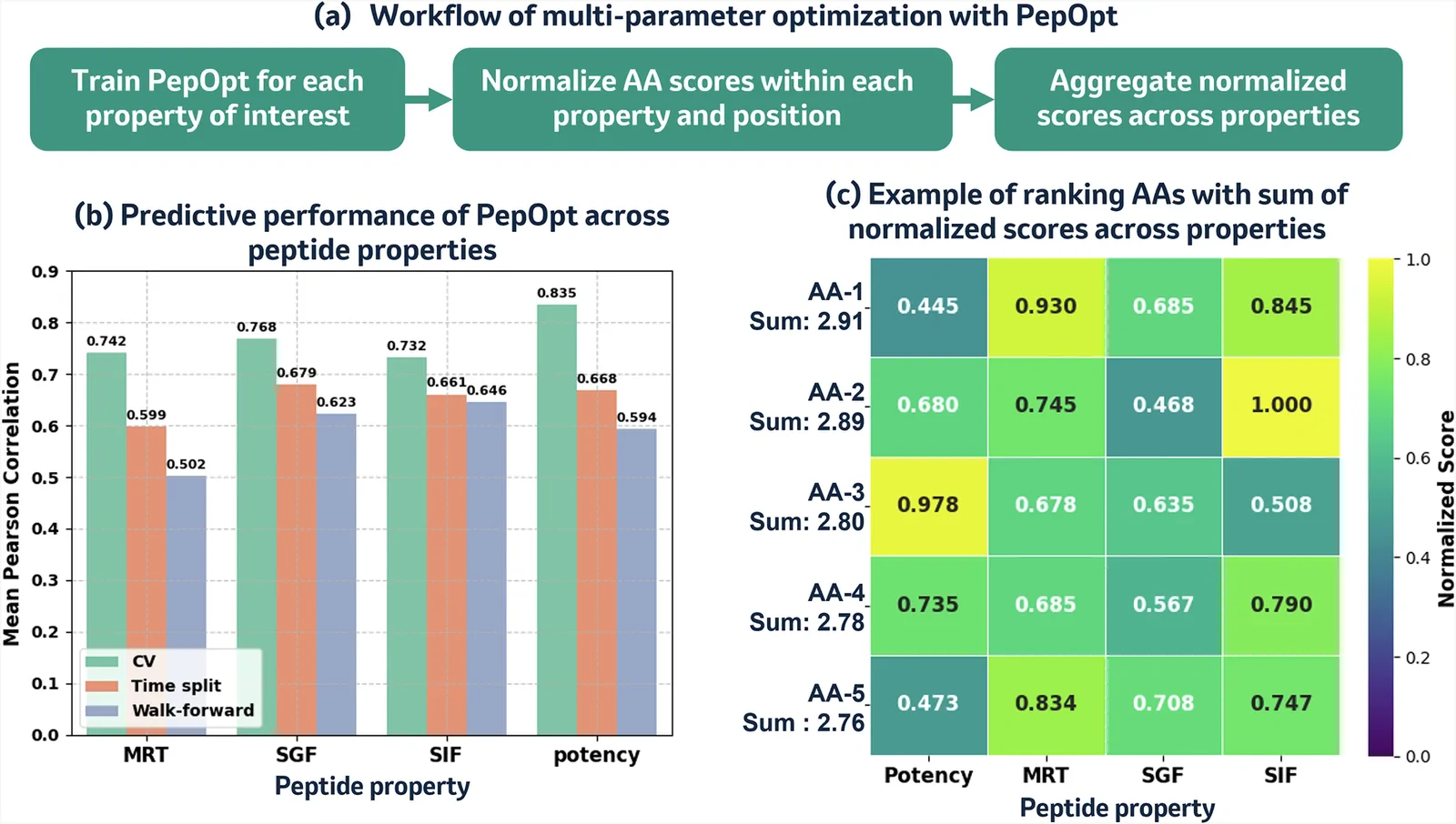
Illustration of applying the PepOpt framework for multi-parameter optimization. **a** Overview of the multi-parameter optimization process, including (1) training PepOpt model for each peptide property of interest, (2) calculating AA scores and normalizing them within each peptide property and position, and (3) aggregating normalized scores across peptide properties. **b** Evaluation of PepOpt for predicting multiple peptide properties, including MRT, SIF, SGF, and potency, using the E-1 series. **c** An example of ranking AAs by the sum of normalized scores across peptide properties. MRT Mean Residence Time, SIF stability in Simulated Intestinal Fluid, SGF stability in Simulated Gastric Fluid.

Although this work has primarily focused on potency prediction, the PepOpt framework can be readily extended to other ADME properties for MPO, as illustrated by the workflow in Fig. 6a.

As a proof of concept, in addition to potency, we evaluated PepOpt’s performance on the E-1 series for predicting several key ADME properties: mean residence time (MRT), stability in simulated intestinal fluid (SIF), and stability in simulated gastric fluid (SGF). This series was selected because it contains the largest number of samples for which these properties have been measured. As shown in Fig. 6b, PepOpt demonstrates decent predictive performance across all evaluated properties in time-split validation (mean PCC ranging from 0.599 to 0.679). Figure 6c provides an example of ranking AAs by the sum of their normalized scores across properties, demonstrating PepOpt’s potential to identify candidate AAs that offer a balanced and favorable profile across multiple pharmacokinetic properties and potency.

## Discussion

In this work, we proposed PepOpt, an interpretable ML framework for peptide SAR and lead optimization by exploring ultralarge peptide design spaces. PepOpt represents both canonical and noncanonical AAs with position-specific molecular fingerprints and employs Bayesian Ridge regression to predict peptide potency. This conceptually simple combination is functionally powerful and addresses key limitations of peptide FW and other QSAR modeling methods. It enables extrapolation to previously unseen ncAAs and computationally efficient prioritization of novel and high-potential candidate peptides from ultralarge design spaces. Beyond prediction, leveraging its interpretable linear model, PepOpt can reveal potency-enhancing substructures and residues to elucidate SAR. Furthermore, PepOpt can be generalized to diverse properties, uncovering property-specific SARs and supporting MPO. Taken together, PepOpt can be a valuable tool for prediction, interpretation, ideation, and ultralarge space exploration to accelerate the rational design of novel peptides.

PepOpt utilizes monomer fingerprints as descriptors. Although many of the existing physical property AA descriptors (e.g., Zscale) can technically be applied to ncAAs, they were originally developed for natural peptides and are rarely calibrated or benchmarked on ncAA-containing sequences. In contrast, fingerprint-based descriptors are data-agnostic: they do not depend on a specific training set or predefined AA library and can be generated for any novel monomer. Furthermore, these fingerprints can provide more chemical granularity than numerical descriptors by partitioning the contributions of ncAAs to specific substructures, which can provide useful insights for future ncAA design.

Secondly, in comparison to complex, nonlinear models (such as RBF-SVR and DL models), PepOpt achieves comparable predictive performance with improved model interpretability and computational efficiency. Linear models can directly use weights from the modeling as “descriptor importances”, i.e., contribution to activity, with explicit positive and negative signs. Thus, PepOpt’s descriptor importances are easily interpretable in terms of the model. In contrast, nonlinear methods, for instance RF, have more complex approaches (e.g., permutation or node purity) for generating importances, and may not produce explicit signs^33^. SHAP (SHapley Additive exPlanations)^34^ can produce importances for any type of model by perturbing the descriptors, but it is computationally expensive. Thus, PepOpt has advantages in scoring peptides and individual amino acids.

Thirdly, compared with the original peptide FW analysis, PepOpt addresses key limitations. The OHE FW model is constrained in two critical ways. (1) It neglects the structural relationships between monomers, and its interpretability is limited to the residue level, meaning it can identify an important AA but cannot explain which part of its structure is responsible for the effect. (2) The FW model cannot generalize beyond its training AAs, excluding ncAAs not within the dataset. Overall, the structure-aware and position-aware approach offered by PepOpt overcomes these limitations, providing both deeper chemical rationale and enabling the prospective screening of larger libraries for novel and high-potential candidate AAs within the model’s substructure-level applicability domain. In the A-2 example shown in Fig. 4, an FW-style OHE model would correctly flag RbMeY at P9 as an important residue but could not explain why; PepOpt, by being inherently structure-aware at the substructure level, further attributes the effect to the R-*β*-methyl substructure. This kind of chemically detailed rationale is what is needed to guide ncAA selection in subsequent rounds of synthesis.

Beyond quantitative prediction, the substructure-level attributions produced by PepOpt can directly support hypothesis-driven ideation. For instance, after identifying the R-*β*-methyl group at P9 as a dominant potency-enhancing motif in series A-2, a natural next step is to enumerate additional ncAAs containing analogous acidic, *β*-methyl, or aromatic motifs for subsequent synthesis campaigns. In this way, the SAR deconvolution produced by PepOpt acts as a proactive ideation tool, highlighting substructures associated with enhanced potency and motivating targeted expansion of the monomer library.

This view also positions PepOpt as complementary to, rather than competing with, 3D structural modeling. Whereas structural modeling can reveal the atomistic interactions that rationalize a given potency difference, PepOpt provides proactive guidance on which residues and substructures should be prioritized for the next round of experimental exploration, allowing the two approaches to feed back into one another.

The design space exploration capabilities of PepOpt are equally important. In practice, peptide ideation does not seek a single optimal sequence, but rather a ranked set of k high-scoring candidates that will be further filtered and prioritized for downstream evaluation and synthesis. Under this setting, selecting only the top-scoring monomer at each position does not fulfill the needs. An alternative strategy is to retain only the top percentage of monomers at each position and enumerate all resulting combinations. However, even this reduced search space remains prohibitively large. For example, retaining 100 monomers per position (approximately the top 2.5% of our library in this work) still yields 100^14^ = 10^28^ possible sequences for a 14-mer peptide, which is computationally infeasible to exhaustively evaluate. This is the reason why selecting top monomers by position, or exhaustive search, can not identify the best peptide sequences, and DP or heuristic sampling methods are required.

PepOpt formulates the optimization problem under an additivity assumption and solves it deterministically using DP. The additivity assumption is critical: without it, the optimal subsequence plus the next best monomer is not guaranteed to remain optimal. For example, if subsequence A1-B1-C1 is predicted as optimal and D1 is individually optimal, a nonlinear model might still favor A1-B1-C1-D2. Thus, nonlinear models, even with monomer-level scores, cannot guarantee global optimality without exhaustive traversal, making deterministic solutions impossible. Despite potentially complex, nonlinear interactions among residues at different positions, our benchmarks show that linear models match the performance of nonlinear models. This suggests that the linearized representation is a reasonable approximation for peptides and captures key information.

PepOpt also extends naturally to MPO. Conventional QSAR workflows typically output separate predictions for multiple endpoints; when the objective is truly multi-target, MPO cannot be posed as a single, tractable optimization problem, and users default to indirect strategies such as Pareto fronts or rule-based filters. In contrast, PepOpt defines a unified monomer score that enables direct optimization with the same methods used for single-target problems. This score is a flexible construct: while it can be implemented as the model weights from ridge regression, it is not restricted to them and can incorporate additional functionality while remaining fully compatible with the DP-based enumeration framework.

Nevertheless, a key limitation of the current workflow lies in the discrepancy between the linear model weights and the true causal effect on the endpoint. While the model effectively captures SAR and provides reasonably accurate predictions, this discrepancy is inevitable because linear regression primarily reflects statistical correlation rather than guaranteeing causal relationships. This issue is exacerbated when the dataset contains biases. For example, a small number of certain high-performing monomers may be repeatedly used as substrates to explore mutations at other positions. Although these monomers are intrinsically favorable, they are frequently paired with less optimal residues, and this can lead to underestimated scores due to dataset bias. More careful monomer scoring schemes could be explored under the same framework.

A second, closely related limitation stems from the unbounded, additive nature of the linear model itself. Few structure–activity relationships in biology are strictly linear, and even where local linearity holds, it does not extend indefinitely: for instance, a protein pocket that is not completely filled by a peptide side chain will favor a larger substituent up to the point of pocket saturation, beyond which the trend plateaus or reverses^35–37^. A linear model has no mechanism to detect such a boundary, so a substructure that was favorable within the training range will continue to receive a favorable weight when extrapolated further, which can over-predict the benefit of ever-larger or ever-more-decorated monomers. More generally, like any machine-learning method, PepOpt is reliable only within its applicability domain^38^. Because the descriptors are atom-based fingerprints rather than residue identities, this domain is defined at the substructure level: predictions are well-supported for ncAAs whose constituent substructures are represented in the training set, and correspondingly less reliable when a candidate monomer introduces substructures that the model has never observed. Our benchmarks (Fig. 3) indicate that, on datasets drawn from ongoing lead-optimization campaigns, the linear model matches the predictive performance of nonlinear alternatives, suggesting that useful local linearity is present in practice; nevertheless, we recommend that PepOpt scores for monomers rich in out-of-domain fragments be treated as hypothesis-generating rather than quantitative, and that triage be performed in combination with orthogonal signals such as 3D structural modeling, chemist judgment, or confirmatory assays.

A conceptually appealing remediation is to replace the linear monomer score with a nonlinear one (for example, a tree-based or kernel-based per-residue score) so that saturation effects can be captured. However, this option is incompatible with the present framework: nonlinear monomer scores break the principle of optimality of DP, and top-*k* enumeration loses its correctness guarantee. Reconciling richer per-residue scoring functions with exploration of ultralarge design spaces is an interesting direction for future work, but falls outside the scope of this study.

In this work, we evaluated different molecular descriptors, peptide representations, modeling methods, and three different train-test splitting types to identify the best configurations in the PepOpt framework. Some of the findings may inform the design of future peptide QSAR models for drug discovery.

First, as shown in Fig. 3, binary FPs performed better than descriptors with counts when used with linear models. This may be because FPs are bounded (zero or one), which can improve stability compared with unbounded numerical descriptors. Another possible reason is that the FPs encode the presence or absence of substructures, and potency is usually closely related to AA structures.

For peptide representations, it can be observed in Fig. 4 that the AA-based representation consistently outperformed the whole-molecule ECFP4 representation with counts when averaged over all datasets. Apparently, partitioning fingerprint information to specific residues adds useful information.

Among different linear regression models, we found that models with L2 regularization, i.e., Ridge and Bayesian Ridge, demonstrated superior performance (Fig. 3). In addition, the optimal L1 ratio values for ElNet ranged from 0.2 to 0.4 (Table S3), indicating a preference for the L2 penalty. These findings suggest the effectiveness of L2 regularization when there are many more features than samples. In contrast, linear models with dimensionality reduction performed poorly, indicating that the full feature space may be required to capture the complex SAR.

Regarding nonlinear models, RBF-SVR showed the best performance. The superior performance of RBF-SVR likely stems from its similarity-based modeling approach. During training, the model identifies a set of critical training samples (support vectors) and learns their corresponding dual coefficients, which represent their importance. For prediction, the RBF kernel first calculates the similarity between a new test peptide and each support vector. The final prediction is then a weighted sum of the support vectors’ dual coefficients, where the weights are these similarity scores. This similarity-based reasoning has proven highly successful in small-molecule QSAR modeling and drug discovery^39–41^. However, the current RBF-SVR implementation has limited model interpretability, as the RBF-kernel is applied at the peptide-level. Future work designing similarity-based peptide QSAR models with enhanced model interpretability could be of interest.

In summary, PepOpt’s substructure-aware, position-specific descriptors, interpretable linear model, and DP-based search collectively address key challenges in ncAA-rich peptide lead optimization—from interpretable SAR elucidation and extrapolation to novel monomers, to deterministic exploration of design spaces exceeding 10^50^ sequences and natural extension to MPO. These capabilities make PepOpt a practical platform for accelerating the rational design of next-generation peptide therapeutics.

## Methods

### PepOpt framework

Here we provide the methodology details for the three components of PepOpt described in this subsection: cyclic peptide alignment, molecular descriptor selection, and linear model selection. To represent cyclic peptides as AA sequences, the peptides were aligned using PepSeA^42^. PepSeA is a tool designed for the alignment of linear, cyclic, and cross-linked peptides with canonical and noncanonical AAs using multiple sequence alignment with fast Fourier transform (MAFFT)^43^. As shown in Fig. 1, PepSeA returns a detailed breakdown by position of aligned peptides for subsequent model training. The substitution matrices used for alignment are calculated based on chemical similarities between thousands of AAs. While the original PepSeA implementation calculated these similarities using computationally intensive 3D descriptors, a later development of our workflow showed that chirality-aware 2D molecular descriptors named atom pair chiral-donor/acceptor pairs (APC-DPC)^44^ provide comparable performance with significantly greater efficiency.

It should be noted that any descriptor that includes an explicit residue position, as is used in PepOpt, is dependent on the alignment of sequences, and this assumes that all peptides in the dataset are sensibly alignable.

PepOpt encodes each peptide by concatenating the molecular descriptors of its constituent AAs position-by-position along the aligned sequence. To enable rapid computation across large AA libraries, including many ncAAs, we focused on descriptors that are both widely used, readily available for ncAAs, and computationally efficient, evaluating four open-source molecular representations: two binary fingerprints and two numerical descriptor sets.

Binary fingerprints: 2048-bit Morgan fingerprints with a radius of 2 and 2048-bit RDKit fingerprints (RDKitFP)^45^. In this paper, we will refer to these as “ECFP4”, since they are equivalent to the ECFP4 descriptors of Rogers, 2010. The Morgan fingerprint is chirality-aware.

Physical property descriptors: Mordred 2D descriptors^46^ and RDKit 2D descriptors (RDKitDesc)^45^. Prior to model fitting, these numerical features were standardized to zero mean and unit variance to improve training stability and ensure comparability across positions.

To establish a baseline aligned with classic additive QSAR modeling, we also included OHE of the AAs, as used in the FW analysis^22,23^. Because these descriptor families are standard in QSAR, we refer readers to the original publications for implementation details and theoretical background.

Because the sequence-based representation of peptides yields far more features than samples, we evaluated six linear models with either regularization or dimensionality reduction: (1) Ridge regression^47^, (2) Lasso regression^48^, (3) Elastic Net regression (ElNet)^49^, (4) Bayesian Ridge regression (B-Ridge)^50,51^, (5) Partial Least Squares regression (PLS)^52,53^, and (6) Principal Component Regression (PCR). The models were implemented with the Python package scikit-learn. In most cases, descriptors were normalized to zero mean and unit variance. For the reader’s convenience, we briefly describe B-Ridge below. For other commonly used linear models, we refer readers to the original publications for implementation details and theoretical background.

B-Ridge refers to Bayesian regression with a spherical Gaussian prior on the weights. In Bayesian regression, the target **y** is assumed follow a Gaussian distribution as follows:

1 $${\bf{y}}| {\bf{X}},{\bf{w}},\alpha \sim {\mathcal{N}}({\bf{X}}{\bf{w}},{\alpha }^{-1}{\bf{I}})$$

where *α* is the noise precision. The prior for the coefficients **w** is also assumed to be Gaussian:

2 $${\bf{w}}| \lambda \sim {\mathcal{N}}(0,{\lambda }^{-1}{\bf{I}})$$

where *λ* is the precision parameter. In B-Ridge, the precision parameters *α* and *λ* are assumed to follow Gamma distributions with non-informative shape and rate of 10^−6^ and are learned from the data by iteratively maximizing the marginal likelihood (or evidence function). Given the precision parameters, the posterior distribution of the weights is as follows:

3 $${\bf{w}}| {\bf{X}},{\bf{y}},\alpha ,\lambda \sim {\mathcal{N}}(\mu ,{\boldsymbol{\Sigma }})$$

where ${\boldsymbol{\Sigma }}={(\lambda {\bf{I}}+\alpha {{\bf{X}}}^{\top }{\bf{X}})}^{-1}$ and *μ* = *α****Σ*X**^⊤^**y**. Here, *μ* is equivalent to the closed-form solution of the Ridge regression with $\frac{\lambda }{\alpha }$ as regularization strength. Given a set of test samples **X**_*t*_, posterior predictive distribution is as follows:

4 $${{\bf{y}}}_{t}| {{\bf{X}}}_{t},{\bf{X}},{\bf{y}},\alpha ,\lambda \sim {\mathcal{N}}({{\bf{X}}}_{t}\mu ,{\alpha }^{-1}{\bf{I}}+{{\bf{X}}}_{t}{\boldsymbol{\Sigma }}{{\bf{X}}}_{t}^{\top })$$

B-Ridge has two main advantages over Ridge: (1) it does not need hyperparameter fine-tuning, and (2) it provides uncertainty measures for the model weights and predictions.

### Model benchmarking

One challenge for benchmarking peptide QSAR models is that peptide datasets often exhibit high molecular similarity, and random splitting can lead to substantial overlap between training and test sets^19,21^. This favors overfitted models and misrepresents predictive performance in real-world deployment. Therefore, we evaluated models using three split methods described below. Data-wise, we prioritize internal datasets with temporal annotations, enabling more rigorous time-based evaluation.

Random cross-validation: Peptides were randomly partitioned into five folds.

Five-fold time split: Peptides were partitioned into five folds by date of registration in the company database. Each fold was used once as the test set, with the remaining four folds serving as the training set. This strategy evaluates generalization to peptides registered at different times while keeping training and test windows comparable in size.

Walk-forward validation: Adapted from time series forecasting, this approach also begins by creating five chronological folds. Starting with the second fold, each fold is used in turn as the test set, and all chronologically earlier folds are aggregated to form the training set. This simulates prospective use, where a model is trained only on data available up to a certain point and then evaluated on the next set of data.

Illustrations for the time split and walk-forward validation can be found in Fig. S1.

Standard nonlinear QSAR models: To assess the predictive performance of PepOpt, we benchmarked it against complex nonlinear models, which are commonly observed to deliver superior accuracy. We evaluated three commonly used nonlinear QSAR models, including RF, SVR, and XGBoost. For these models, we evaluated two distinct peptide representations. The first was a position-wise concatenation of ECFP4 descriptors for each AA (noted as aa), identical to the representation used in PepOpt. The second was a count-based ECFP4 computed from the entire peptide molecule (noted as pep). The standard models were implemented with the Python package *scikit-learn* and *xgboost*.

CheMeleon: Besides the above nonlinear models, graph convolutional networks (GCNs) such as ChemProp^54^ are another type of commonly used model in practice. It is challenging to properly train a GCN model from scratch with the given dataset sizes. Therefore, we chose CheMeleon^55^ as the representation of such a model type. It was pre-trained on millions of small molecules to learn rich molecular representations from low-noise molecular descriptors, with predictivity proven to outperform baselines like RF and ChemProp in benchmark studies. We implemented the model with the pre-trained model weights following its documentation (CheMeleon Foundation Finetuning—Chemprop 2.2.1 documentation).

Pepland: Pepland^20^ is a pretraining framework designed to learn informative representations from both natural and noncanonical peptides. It employs an adaptive fragmenting strategy (AdaFrag) combined with a multi-view heterogeneous graph neural network to capture multilevel structural representations of peptide sequences.

In this work, the pretrained Pepland model was used to generate embeddings from peptide SMILES. To better adapt the model to ncAAs present in proprietary data, we performed an additional round of pretraining using proprietary datasets, resulting in improved performance compared with the publicly released checkpoint. Starting from the public checkpoint, the model was further pretrained on approximately 20,000 proprietary peptide sequences, together with the ncAA-containing peptides provided by the original publication. The same tokenization scheme (AdaFrag, 258 tokens) and hyperparameters recommended in the paper were used.

300-dimensional embeddings were generated using gated recurrent unit (GRU) pooling, with all model weights frozen. MLP hyperparameters are tuned with the same protocol as other models. During downstream evaluation, Pepland-based models exhibited extreme outlier predictions that disproportionately affected fold-level averages. To better reflect overall model performance, Pepland prediction outliers greater than 15 or less than 0 for pEC50 (nM) were censored and replaced with the corresponding fold mean prediction.

We fine-tuned all models except B-Ridge and CheMeleon. B-Ridge learns its optimal hyperparameters from the training data, and CheMeleon is a pre-trained model and was used with the same model architecture as provided, without additional hyperparameter-tuning.

For each peptide representation, we utilized the Python package Optuna^56^ to fine-tune the hyperparameters on three randomly selected peptide series across three different splitting types. For each splitting type, the optimal hyperparameters were determined by minimizing the mean squared error averaged over splitting folds (50 trials for linear models and 100 trials for nonlinear models). The resulting per-series, per-splitting type optimal values were then averaged to obtain a single set of final hyperparameters for each model and peptide representation. Tables S3 and S4 report the optimal hyperparameters for the linear and nonlinear models, respectively.

We evaluated the predictive performance using *R*^2^ scores, Spearman’s rank correlation coefficient (*ρ*), and Pearson correlation coefficient (PCC). These performance metrics are calculated as follows:

5 $$\begin{array}{l}{R}^{2}=1-\frac{\mathop{\sum }\nolimits_{i = 1}^{n}{({y}_{i}-{\hat{y}}_{i})}^{2}}{\mathop{\sum }\nolimits_{i = 1}^{n}{({y}_{i}-\bar{y})}^{2}}\\ \rho =1-\frac{6\mathop{\sum }\nolimits_{i = 1}^{n}{d}_{i}}{n({n}^{2}-1)}\\ \,{\mathrm{PCC}}\,=\frac{\mathop{\sum }\nolimits_{i = 1}^{n}({y}_{i}-{\bar{y}})({\hat{y}}_{i}-{\bar{\hat{y}}})}{\sqrt{\mathop{\sum }\nolimits_{i = 1}^{n}{({y}_{i}-{\bar{y}})}^{2}\mathop{\sum }\nolimits_{i = 1}^{n}{({\hat{y}}_{i}-{\bar{\hat{y}}})}^{2}}}\end{array}$$

where *n* is the number of test samples, *y*_*i*_ and ${\hat{y}}_{i}$ are the observed and predicted values, respectively, $\bar{y}$ and $\bar{\hat{y}}$ are the mean observed and predicted values, respectively, and *d*_*i*_ is the difference between the ranks of the observed and predicted values. *R*^2^ indicates how well the model can predict the exact target values. Spearman’s *ρ* assesses the monotonic relationship between the observed and predicted responses. It will be close to unity if the predicted and observed responses have similar ranks. PCC, on the other hand, measures the linear relationship between the predicted and observed responses.

### Efficiently explore ultralarge peptide design spaces with dynamic programming

To illustrate the difference of the PepOpt framework with heuristic sampling methods, we performed a comparison between DP-based enumeration and GA search, under the identical training set and target design spaces. In the GA implementation, the same Bayesian Ridge model was trained as the acquisition model and used to score generated peptide sequences. We started from the peptide with the lowest measured activity (*pEC*50 = 5.94(nM)), representing the sub-optimal hit before entering the lead-op phase. In each generation, offspring sequences are generated by replacing monomers with random ones from the library at random positions. Cross-over, insertion, and deletion are not considered, and the peptides were kept at the same length throughout the search. The *k* highest-scored offspring were carried into the next generation. There is always a trade-off between search depth and runtime for GA, and we adopted a fixed computational budget: the algorithm was run for 50 generations, with 100 offspring generated per generation by mutating two randomly selected positions, and the top 20 sequences were propagated to the next generation. All tasks comparing computational cost were done with a single CPU core of the same type with sufficient memory.

### Multi-parameter optimization (MPO)

The PepOpt framework is inherently compatible with MPO. In peptide lead optimization cycles, achieving desirable physicochemical properties is as critical, if not more so, than potency. We want to assign a goodness score to a specific AA at a specific residue position, based on one or more criteria other than potency. PepOpt addresses this by assigning monomer scores based on multiple activities. Rather than fitting a single model for potency, we can fit multiple linear models for different activities (e.g., stability, clearance, etc.) and take the weighted average of the model weights as an MPO monomer score to reflect multiple properties comprehensively. This modification only applies to monomer score calculation, without affecting other parts of this framework. By finding the best monomers, we can suggest peptides with the best predicted overall properties.

## Supplementary information

Supplementary Information

## Data Availability

Our work focuses on peptide lead optimization, particularly in design spaces with a high proportion of ncAAs. Existing public datasets fall short in reflecting the chemical spaces and tasks we intended to address. For this reason, we relied on proprietary datasets derived from active production pipeline projects to demonstrate and evaluate our framework. Unfortunately, due to IP restrictions, we cannot provide all the datasets used for reproducing the model benchmarking results. Instead, we provided the descriptive statistics and would encourage readers to examine the framework's efficiency on other projects.

## Code availability

To facilitate the reproducibility and extension of this work, we have made an open-sourced implementation available at GitHub https://github.com/MSDLLCpapers/Pepopt. An example dataset for demonstrating the workflow is provided in the same repository, whose data is extracted from patent WO2024215820. The experiment scripts for producing the results are also provided in the same repository.
