# Supplementary material for: Interpretable machine learning framework for peptide lead optimization in ultralarge design spaces
Source: NPJ Drug Discov. 2026 Oct 12;3:45. doi: 10.1038/s44386-026-00069-y (PMC13658789; doi:10.1038/s44386-026-00069-y)
Supplement: Supplementary file 1 [file 44386_2026_69_MOESM1_ESM.pdf]

Supplementary Information for PepOpt: An interpretable machine learning framework for peptide lead optimization in ultralarge design space

S1 Supplementary Tables

Table S1: Summary of the peptide series used in this study

Series ID	Length	Num. peptides	Num. peptides (% ncAA>50)	Num. ncAAs	Molecular weight	Num. atoms
A-1	16	775	393	230	2041±54	146±4
A-2	14	879	868	373	1985±56	144±4
A-3	17	456	100	188	2078±58	149±4
B-1	17	1491	1440	456	2268±69	159±5
B-2	15	511	264	249	1906±66	136±5
B-3	15	415	376	220	2121±50	152±4
C-1	14	899	712	322	2034±100	148±7
C-2	15	687	616	322	2077±76	150±6
C-3	17	689	31	255	2062±95	148±7
D-1	14	504	504	225	2064±47	149±3
E-1	14	2013	2013	427	2133±113	153±8

Table S2: Comparing PepOpt against non-linear models in peptide potency prediction

Model	CV			Time Split			Walk-forward		
	R^2	Spearman	PCC	R^2	Spearman	PCC	R^2	Spearman	PCC
PepOpt	0.74 ± 0.11	0.85 ± 0.07	0.86 ± 0.07	0.28 ± 0.33	0.67 ± 0.15	0.67 ± 0.14	0.16 ± 0.33	0.61 ± 0.14	0.61 ± 0.14
RBF-SVR aaECFP[1]	0.76 ± 0.13	0.87 ± 0.07	0.88 ± 0.08	0.30 ± 0.40	0.69 ± 0.15	0.68 ± 0.15	0.21 ± 0.34	0.61 ± 0.15	0.62 ± 0.14
RF aaECFP	0.71 ± 0.12	0.83 ± 0.09	0.85 ± 0.07	0.13 ± 0.32	0.56 ± 0.18	0.54 ± 0.18	0.05 ± 0.32	0.49 ± 0.17	0.48 ± 0.18
XGBoost aaECFP	0.74 ± 0.13	0.85 ± 0.08	0.86 ± 0.08	0.15 ± 0.50	0.61 ± 0.16	0.60 ± 0.17	0.06 ± 0.46	0.56 ± 0.14	0.55 ± 0.17
RBF-SVR pepECFP	0.70 ± 0.11	0.82 ± 0.07	0.84 ± 0.06	0.07 ± 0.50	0.55 ± 0.16	0.55 ± 0.16	-0.11 ± 0.69	0.45 ± 0.14	0.46 ± 0.15
RF pepECFP[2]	0.70 ± 0.11	0.82 ± 0.07	0.84 ± 0.07	0.09 ± 0.36	0.55 ± 0.18	0.54 ± 0.20	-0.00 ± 0.38	0.48 ± 0.14	0.48 ± 0.18
XGBoost pepECFP	0.71 ± 0.11	0.83 ± 0.08	0.85 ± 0.06	0.15 ± 0.40	0.58 ± 0.17	0.58 ± 0.18	0.06 ± 0.38	0.51 ± 0.14	0.52 ± 0.15
Chemeleon (MPNN)	0.67 ± 0.18	0.80 ± 0.11	0.81 ± 0.14	0.13 ± 0.43	0.57 ± 0.16	0.57 ± 0.16	0.05 ± 0.32	0.50 ± 0.16	0.50 ± 0.19
Pepland +[3] 2-layer MLP	0.14 ± 0.28	0.44 ± 0.19	0.45 ± 0.20	-0.71 ± 0.89	0.18 ± 0.22	0.15 ± 0.21	-1.32 ± 1.70	0.08 ± 0.19	0.05 ± 0.15

[1]aaECFP: position-wise concatenation of ECFP4 descriptors for each AA, identical to the representation used in PepOpt.
[2]pepECFP: the count-based ECFP4 computed for the whole peptide.
[3]Embeddings are generated with frozen model weights. Outliers in Pepland MLP predictions are removed.

Table S3: Optimal hyperparameters for the linear models

Model	Hyperparmeter	Optimal values					Hyperparameter explanation
		ECFP4	RDKitFP	RDKDesc	Mordred	OHE	
Ridge	alpha	13.933	141.031	609.827	896.781	2.021	Control the L2 penalty
Lasso	alpha	0.003	0.005	0.018	0.018	0.004	Control the L2 penalty
ElNet	alpha	0.004	0.009	0.080	0.074	0.003	alpha*l1_ratio controls L1 penalty
	l1_ratio	0.403	0.323	0.294	0.340	0.196	alpha*(1-l1_ratio) controls L2 penalty
PLS	n_components	5	8	6	6	2	Number of components to keep
PCR	perc_comp	0.270	0.231	0.155	0.152	0.241	Number of PCs to keep is calculated as int(perc_comp*num_samples)

Table S4: Optimal hyperparameters for the non-linear models

Model	Hyperparameter	Optimal values for different descriptors		Hyperparameter explanation
		aaECFP	pepECFP	
RF	max_depth	17	18	The maximum depth of the tree
	min_samples_leaf	2	2	The minimum number of samples for a leaf node
	min_samples_split	5	4	The minimum number of samples to split an internal node
	n_estimators	296	250	The number of trees
XGBoost	learning_rate	0.165	0.124	Learning rate
	max_depth	6	4	The maximum depth of the tree
	n_estimators	248	326	The number of trees
	reg_alpha	0.743	0.085	L1 regularization term on leaf weights
	reg_lambda	1.950	4.281	L2 regularization term on leaf weights
RBF-SVR	C	71.98	33.13	1/C controls the regularization strength
	epsilon	0.086	0.111	No penalty for points predicted within a distance epsilon
	gamma	scale	auto	Kernel coefficient for the RBF kernal

Table S5: Optimal hyperparameters for Pepland - MLP

	MLP	
Hyperparmeter	Optimal values	Explanation
learning_rate_init	0.006148	Constant learning rate used in training
hidden_layer_sizes	(300, 322)	Number of neurons in the two hidden layers

Table S6: Number of used AAs in the series of A-2 across positions

P1	P2	P3	P4	P5	P6	P7	P8	P9	P10	P11	P12	P13	P14
40	10	18	61	57	21	12	5	97	36	69	33	21	55

S2 Supplementary Figures

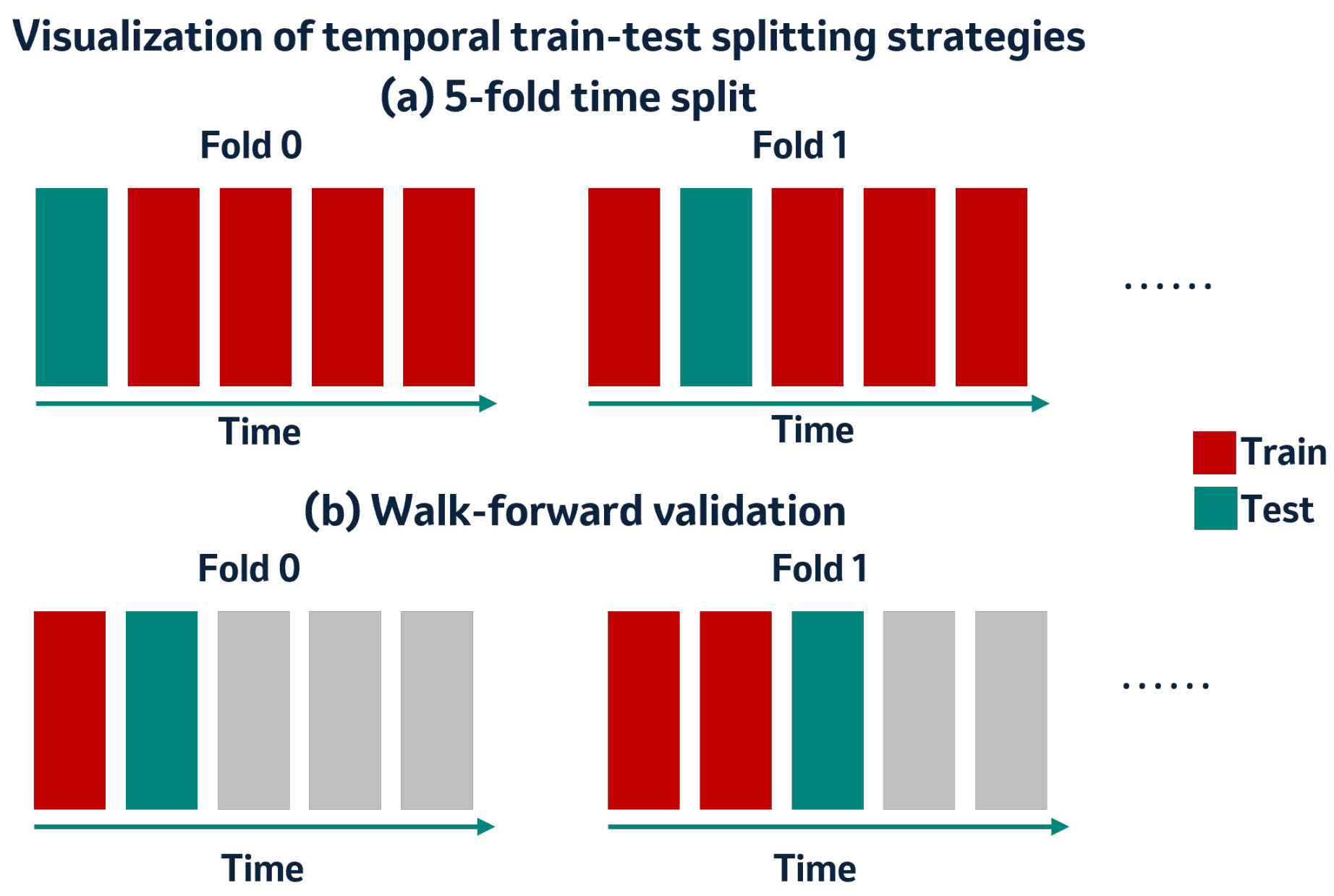

Figure S1: Visualization of time split and walk-forward validation.

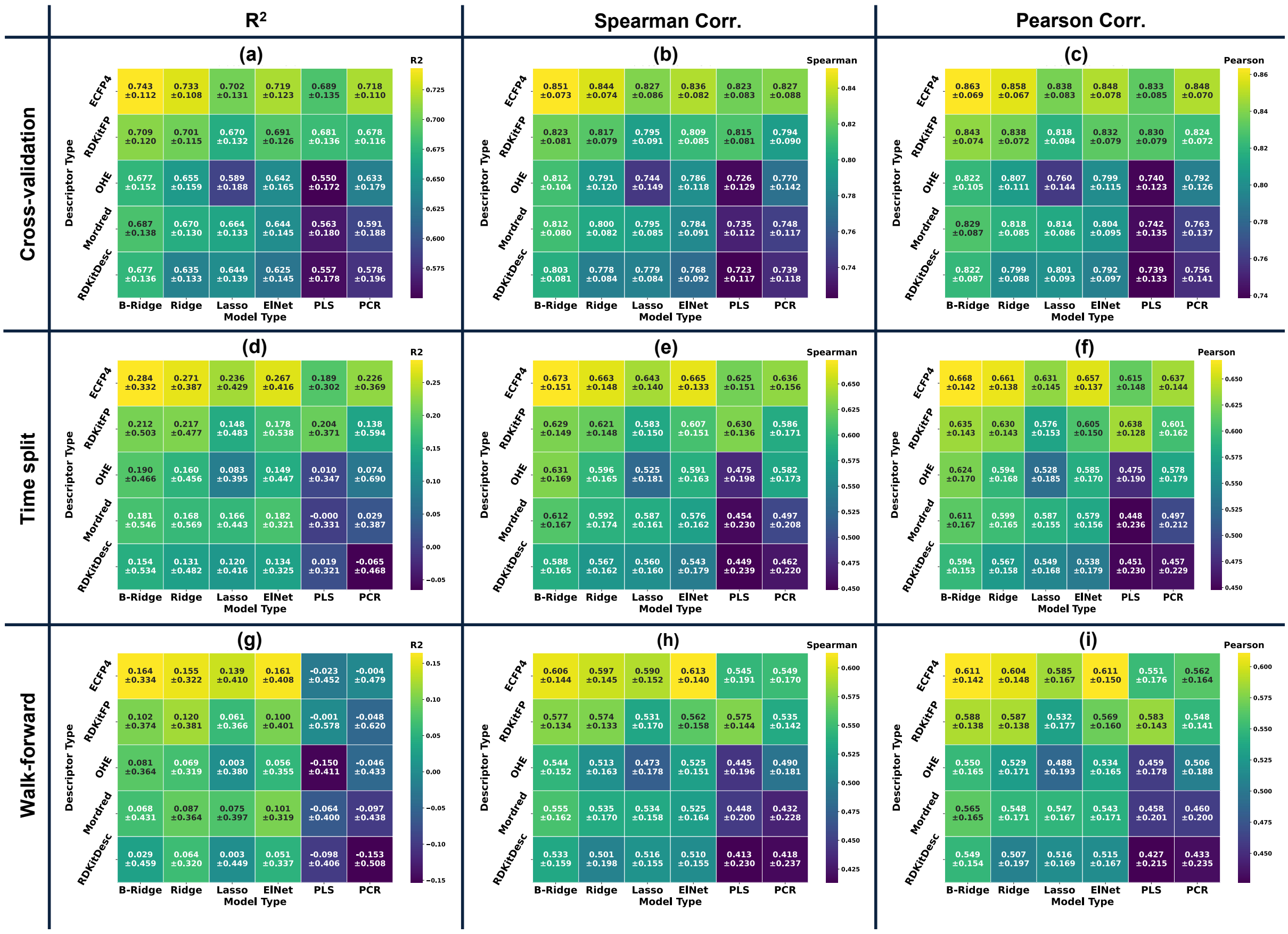

Figure S2: Evaluation of descriptor–model combinations for predicting macrocyclic peptide potency with three different train-test splitting types: 5-fold cross-validation (top row), 5-fold time split (middle row), and walk-forward validation (bottom row). The heatmaps visualize averaged performance over split folds and peptide series for three metrics: R2 (left column), Spearman's rank correlation (middle column), and Pearson correlation (right column). Cell annotations report the mean, standard deviation, and performance rank within each splitting types and performance metrics. Abbreviations: OHE, one-hot encoding; RDKitDesc, RDKit descriptors; B-Ridge, Bayesian Ridge; ElNet, Elastic Net; PLS, partial least squares; PCR, principal component regression.

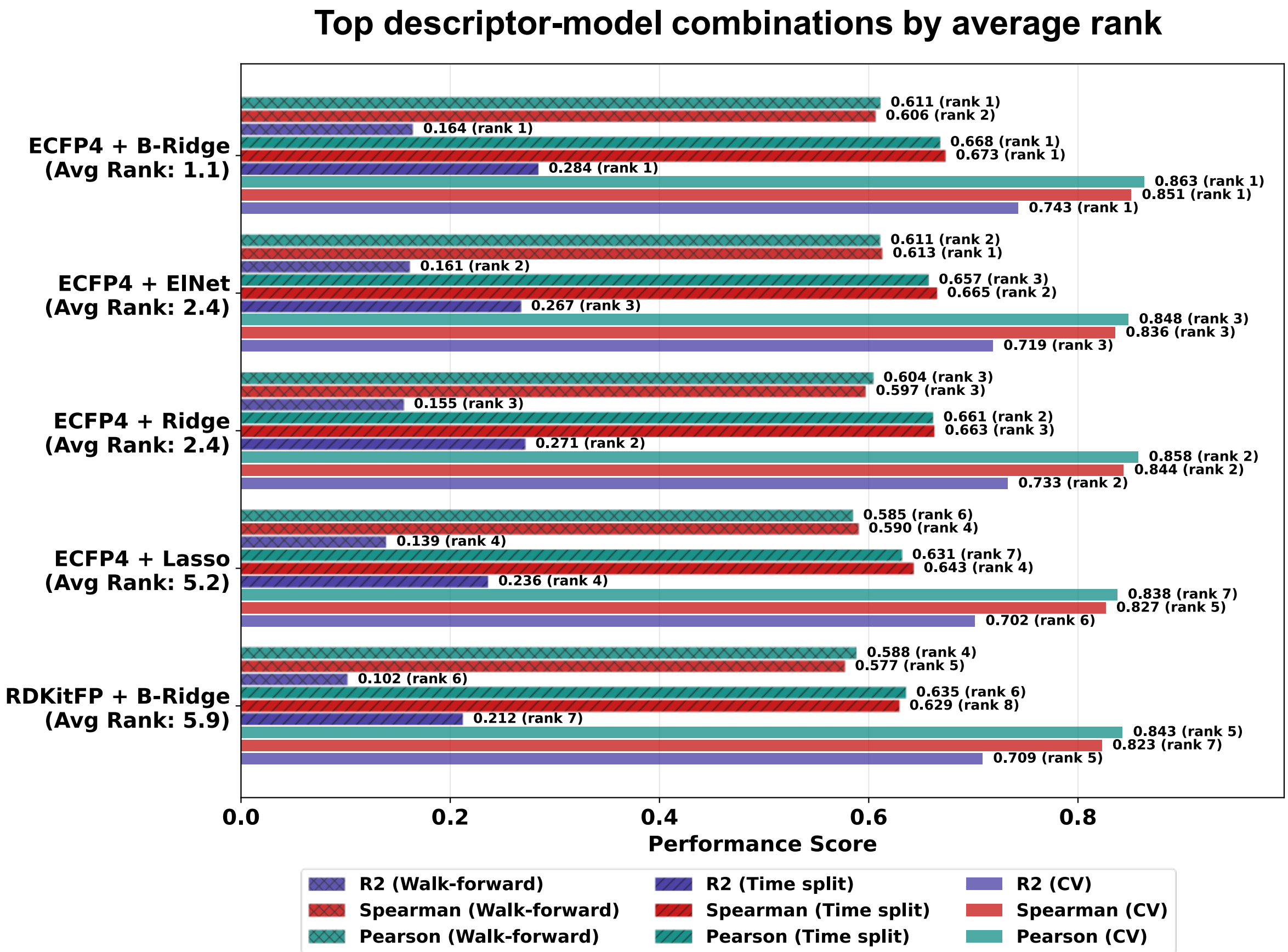

Figure S3: Performance of the top five descriptor–model combinations, selected by lowest average rank across split types and performance metrics. Bars show mean values aggregated over all folds and peptide series for each split type and performance metric.

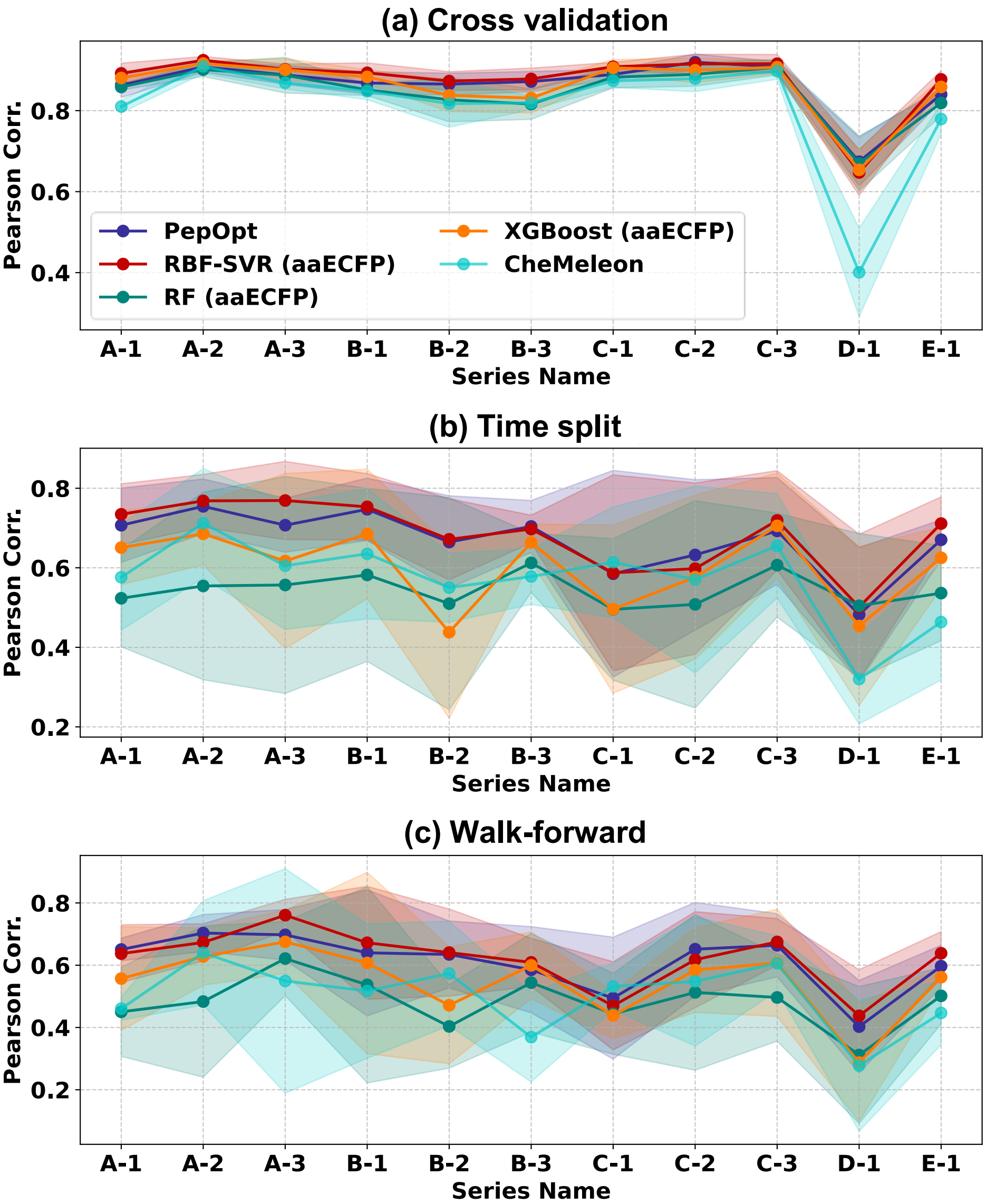

Figure S4: Series-wise performance of PepOpt versus non-linear models (RBF-SVR, RF, XGBoost, and CheMeleon). Bar plots show averaged Pearson correlations across split folds for: (a) 5-fold cross-validation, (b) 5-fold time split, and (c) 5-fold walk-forward validation. Error bars indicate standard deviations over folds.

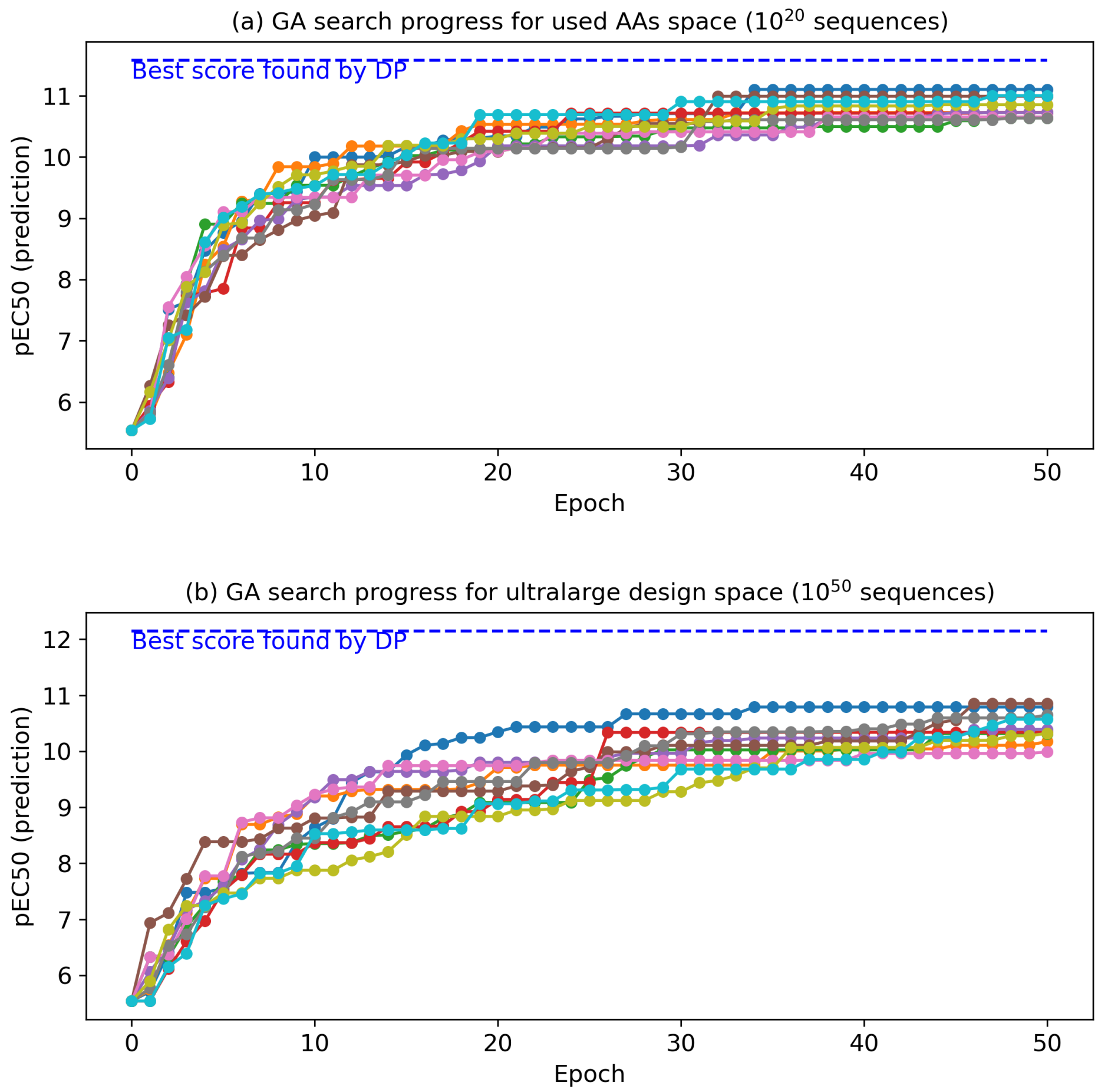

Figure S5: Evolution progresses of 10 random GA search campaigns on two design spaces respectively.

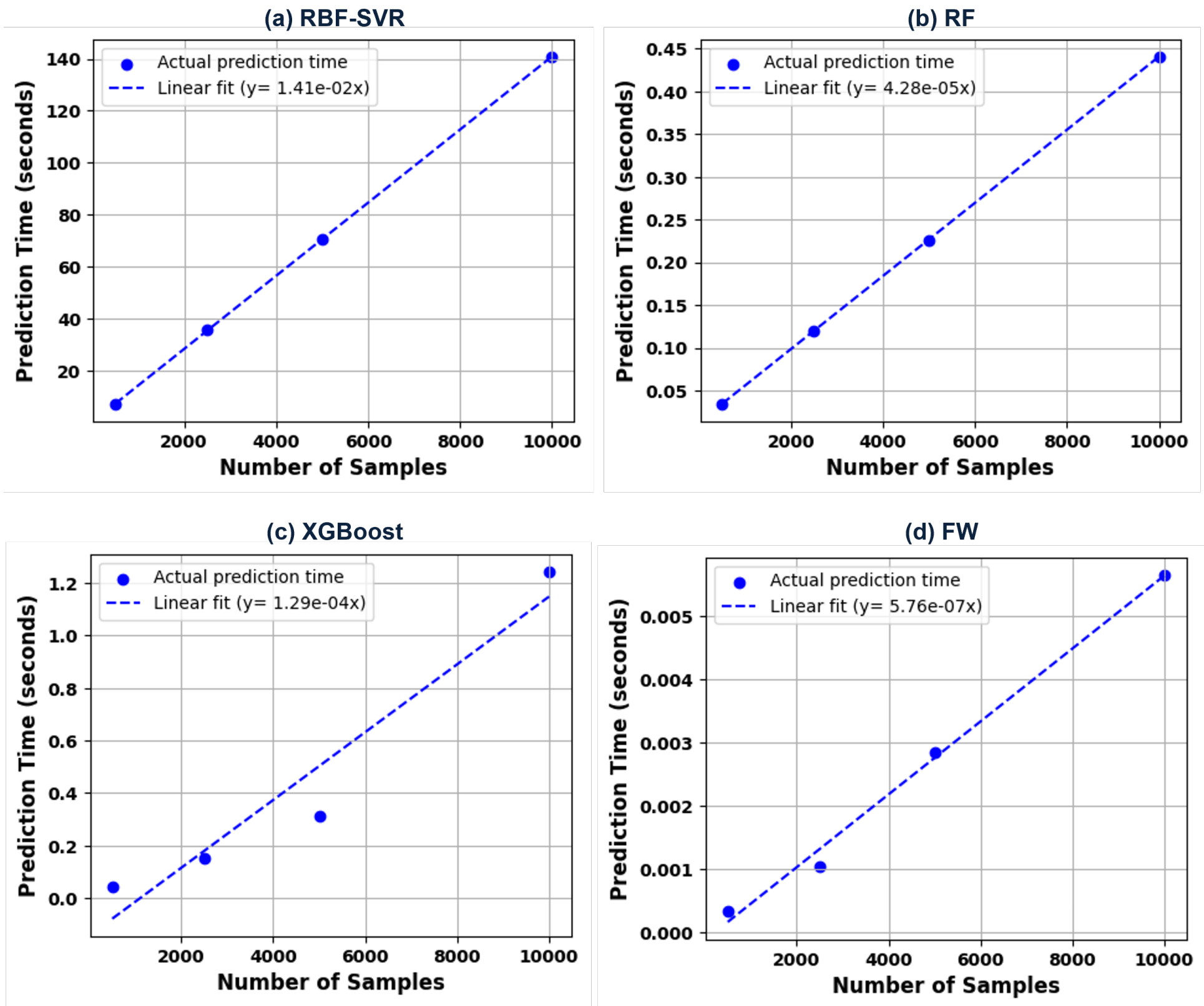

Figure S6: Prediction time as a function of sample size for RBF-SVR, RF, XGBoost, and FW models. Dashed lines represent linear fits used to extrapolate the time complexity for screening an ultralarge peptide design space.
